# lncRNA FENDRR Predicts Adverse Prognosis and Regulates the Development of Esophageal Squamous Cell Carcinoma Through Negatively Modulating miR-495-3p

**DOI:** 10.5152/tjg.2025.24350

**Published:** 2025-05-20

**Authors:** Yangyang Xue, Ran Yang, Ping Gong, Hongjuan Zhu

**Affiliations:** Department of Internal Medicine, Jiangsu Cancer Hospital, Jiangsu Institute of Cancer Research, the Affiliated Cancer Hospital of Nanjing Medical University, Jiangsu, China

**Keywords:** Biomarker, ceRNA, FENDRR, long non-coding RNA, tumor progression, esophagus cancer

## Abstract

**Background/Aims:**

Esophageal squamous cell carcinoma (ESCC) is a major subtype of esophageal carcinoma and is highly prevalent in China. Identification of effective biomarkers could benefit ESCC management and therefore improve clinical outcomes. Evaluating the expression and significance of long non-coding RNA Fetal-lethal non-coding developmental regulatory RNA (FENDRR) in ESCC aims to provide a biomarker candidate for ESCC.

**Materials and Methods:**

This study enrolled 117 ESCC patients and collected tissue samples. The expression of FENDRR in collected samples was analyzed by polymerase chain reaction. The Chi-square, Kaplan–Meier, and Cox analyses were performed to reveal its clinical value. In ESCC cells, FENDRR was regulated by cell transfection, and its effect on cell growth and motility was evaluated.

**Results:**

FENDRR was downregulated in ESCC and was associated with large tumor size, poor differentiation, late TNM stage, positive lymph node metastasis, and adverse development-free survival of ESCC patients. FENDRR acted as an adverse indicator for the prognosis of ESCC patients. miR-495-3p was negatively regulated by FENDRR. Overexpressing FENDRR significantly suppressed ESCC cell growth and metastasis, while miR-495-3p reversed these effects.

**Conclusion:**

Downregulated FENDRR in ESCC predicted the malignant development and adverse prognosis of ESCC patients. FENDRR served as a tumor suppressor of ESCC by modulating miR-495-3p.

**Main Points**

- Downregulated FENDRR predicts the severity of esophageal squamous cell carcinoma (ESCC)..
- FENDRR served as an independent prognostic biomarker for ESCC.
- FENDRR suppressed ESCC cell progression by modulating miR-495-3p.

## Introduction

Esophageal carcinoma is a common malignant tumor of the digestive system and tops the mortality of malignant tumors.^1^ There are significant differences in the occurrence and prevention of various histological types of esophageal carcinoma, with esophageal squamous cell carcinoma (ESCC) accounting for a huge percentage in Asia. Due to unclear pathogenesis, although multidisciplinary and comprehensive treatment has greatly improved, the incidence of ESCC has increased rapidly in the past decades.^2^^,^^3^ The risk factors for ESCC were demonstrated to be associated with heavy drinking, smoking, poor oral health, and pickled vegetables, with the onset of ESCC involving the complex processes of multiple stages and interactions.^4^^,^^5^ Surgical resection is an effective treatment for ESCC, but it is only suitable for patients at an early stage. Therapeutic strategies for patients at advanced stages are still limited.^6^^,^^7^ The activation of oncogenes and the inactivation of anti-oncogenes have been suggested to play vital roles in the molecular mechanism of ESCC progression.^8^ Therefore, the genetic and epigenetic changes of ESCC patients have become current research hot points, which could help identify novel molecular targets for monitoring ESCC development, exploring better management, and therefore improving patients’ survival.

Non-coding RNAs (ncRNAs) have been demonstrated to occupy an important position in the human genome. The regulation of ncRNAs is also involved in epigenetics, where long non-coding RNAs (lncRNAs) attract special attention. With the development of sequencing technology, the role of lncRNAs in tumor progression has been noticed.^9^ Although lncRNAs cannot code for proteins, they have been evidenced to regulate gene expression networks through chromatin modification, transcription and post-transcriptional regulation, and other pathways. The effect of lncRNAs, especially the dysregulated and progression-related lncRNAs, on the occurrence and progression of malignant tumors has also been disclosed. Recent studies have attempted to construct lncRNA signatures related to ESCC prognosis and cancer development, identifying a series of candidate biomarkers, involving lncRNA FENDRR (FENDRR).^10^^,^^11^ Long non-coding RNA Fetal-lethal non-coding developmental regulatory RNA (FENDRR) has been identified as a cancer-related lncRNA that inhibited colon cancer progression, mediated gastric cancer cellular processes, and regulated the tumor immune microenvironment of non-small cell lung cancer.^12^^-^^14^ Whether FENDRR could serve as a biomarker of ESCC monitoring disease development and predicting patients’ outcomes remains unknown.

In this study, the expression of FENDRR was estimated in ESCC. The significance and potential of FENDRR in patients’ prognosis and development-related cellular processes were also assessed, aiming to identify a novel biomarker candidate for ESCC. Additionally, the regulatory mechanism underlying the function of FENDRR was also investigated. Competing endogenous RNA (ceRNA) theory has been accepted as the major mechanism underlying the function of lncRNAs and circRNAs, where lncRNAs are considered to negatively regulate functional miRNAs and further mediate disease development. Previously, FENDRR was revealed to sponge miR-424-5p, displaying its tumor suppressor role in colorectal cancer, and various miRNAs were identified to mediate its function in tumor progression.^15^^-^^19^ According to the prediction of related miRNAs of FENDRR from online databases, miR-495-3p was identified as a sponge of FENDRR. Moreover, miR-495-3p was disclosed to serve as a target of FAM83A-AS1, mediating the regulation of tumor progression of esophageal cancer.^20^ Hence, the involvement of miR-495-3p was hypothesized as the potential mechanism underlying the effect of FENDRR.

## Materials and Methods

### Inclusion and Exclusion Criteria

This study had been approved by the Ethics Committee of Jiangsu Cancer Hospital, Jiangsu Institute of Cancer Research, the Affiliated Cancer Hospital of Nanjing Medical University (No. 2016-002-018), and obtained informed consent from all participants. One hundred and seventeen individuals diagnosed with ESCC by biopsy and who received radical tumor resection at Jiangsu Cancer Hospital, Jiangsu Institute of Cancer Research, the Affiliated Cancer Hospital of Nanjing Medical University were enrolled from June 2016 to July 2018 according to the following criteria:

Inclusion: 1) primarily diagnosed with ESCC; 2) had never received any anti-cancer treatments before diagnosis; 3) the clinical records were complete.

Exclusion: 1) patients under 18 years of age; 2) patients with other malignant tumors or other immune system diseases; 3) pregnant or lactating patients.

### Sample Collection

Tumor tissues were collected during surgery, along with adjacent normal tissues. The distance between normal tissues and tumor tissues was at least 5 cm. The tumor tissues were confirmed to have not invaded the surrounding tissues by at least 2 pathologists. Collected tissues were stored in liquid nitrogen and frozen in a −80°C refrigerator for long-term storage.

### Follow-Up Survey

Patients were followed up for 5 years by telephone or through outpatient review. The endpoint events were defined as recurrence, malignant development, and ESCC-related deaths. The follow-up data were analyzed by Kaplan–Meier and Cox regression analyses.

### Cell Culture

Human-sourced ESCC cell lines, KYSE-150, ECA-109, TE10, and YES-2, and Het-1A (a human normal esophageal epithelial cell) were incubated in 10% fetal bovine serum (FBS) (Invitrogen, USA)-supplemented RPMI1640 culture medium (Invitrogen, USA) at 37°C with 5% CO_2_. The culture medium was replaced with fresh medium every 1-2 days. Cells were available for the following experiments when the confluence reached 80%.

### Cell Transfection

The miR-495-3p was predicted from the lncRNASNP 3.0 database to bind with FENDRR. Cells were incubated with pcDNA 3.1-FENDRR- or miR-495-3p mimic-containing culture medium at 37°C with 5% CO_2_ for 6 hours, then the culture medium was replaced with 10% FBS-containing RPMI 1640 culture medium and incubated for another 2 days. The sequence of miR-495-3p mimic was 5’- AAACAAACAUGGUGCACUUCUU-3’, while the overexpression of FENDRR was conducted through cloning the polymerase chain reaction (PCR) products into the pcDNA 3.1 vector (Invitrogen, USA). The primer sequences were summarized in the corresponding methods section.

### Total RNA Extraction

Tissues were cut up and prepared as homogenate in the Trizol reagent (Invitrogen, USA), while cells were also lysed with Trizol reagent (Invitrogen, USA) after washing with PBS (Sigma-Aldrich, USA). The mixture was centrifuged and the supernatant was collected. Then, a 0.2-fold volume of chloroform (Sigma-Aldrich, USA) was added and centrifuged (12 000 rpm for 10 minutes) after 2-min incubation. The aqueous phase was further mixed with isopropyl alcohol (Sigma-Aldrich, USA) and incubated for 10 minutes at room temperature. Total RNA was isolated and washed with 75% ethanol (Sigma-Aldrich, USA), and recombinant DNase I (Takara, Japan) was used to remove genomic DNA. The OD260/OD280 was detected with NanoDrop 2000 (Thermo Scientific, USA) to assess the concentration and purity of isolated RNA. The ratio of OD260/280 ranged from 1.8 to 2.2, indicating the qualified RNA.

### Real-Time Polymerase Chain Reaction

The cDNA was generated through reverse transcription using a High-Capacity complementary DNA Reverse Transcription Kit (for FENDRR, Applied Biosystem, USA) and a miRcute miRNA cDNA kit (for miR-495-3p, Tiagen, China). Then, FENDRR and miR-495-3p levels were analyzed on the 7500 PCR system (Applied Biosystem, USA) with the SYBR Green Mix (Applied Biosystem, USA). The thermal cycles were conducted at 37°C for 5 minutes, and then at 94°C for 5 minutes, followed by 50 cycles of 90°C for 15 s, 60°C for 60 s, and cooling at 50°C for 30 s. The 2^-∆∆Ct^ method was used for the calculation with GAPDH and cel-miR-39 as internal references. The primer sequences were: FENDRR forward 5’-TAAAATTGCAGATCCTCCG-3’, FENDRR reverse 5’-AACGTTCGCATTTAGC-3’; miR-495-3p forward 5’-GCGAAACAAACATGGTGC-3, miR-495-3p reverse 5’-GCAGGGTCCGAGGTATTC-3’; GAPDH forward 5’-GCCTTCTCTTGTGACAAAGTG-3’, GAPDH reverse 5’-CTTCCCATTCTCAGCCTTG-3’; cel-miR-39 forward 5’-CGTATGAGCGTCACCGGGTGTAAATCA-3’.

### Dual-Luciferase Reporter Assay

The binding sites between FENDRR and miR-495-3p were predicted from the lncRNASNP 3.0 database. The wild-type and mutant-type vectors of FENDRR were constructed by cloning the corresponding sequences into the pGL3 plasmid (Promega, USA). Cells were co-transfected with the established vectors and miR-495-3p mimic, inhibitor, or negative controls according to the cell transfection. Then, the luciferase activity of FENDRR was measured using a luciferase reporter system with Renilla as the internal reference.

### Cell Proliferation Assay

Cells were seeded into 96-well plates with 5 duplicate wells for each group. Cells were maintained in FBS-containing culture medium, and a total of 100 μL cell-free culture medium was added in a ring around the 96-well plates to prevent evaporation. Cell counting kit-8 (CCK8) reagent (Beyotime, China) was added to each well after a period of incubation at 37°C. After incubation for another 2 hours, the plates were analyzed with a microplate reader for OD450.

### Cell Metastasis Assay

Cell suspension was prepared in an FBS-free culture medium, and cells were seeded into the upper chamber of 24-well Transwell plates (Corning, USA). The upper chamber was pre-coated with Matrigel (Corning, USA) diluted with an FBS-free culture medium in the invasion assay. The bottom chamber was filled with a complete culture medium. After a 2-hour incubation at 37°C, the chambers were washed with PBS twice. Cells were counted under a microscope from 5 random fields after fixing and staining.

### Statistical Analysis

All data were presented as mean ± SD (n = 3) and analyzed by SPSS 26.0 software (IBM SPSS Corp.; Armonk, NY, USA). Differences were compared using Student’s *t*-test and one-way ANOVA (*P* < .05). The clinical data were analyzed with the Chi-square test to estimate the association of FENDRR with patients’ disease conditions. Kaplan–Meier and multivariate Cox regression analyses were performed for the analysis of follow-up data and to identify prognostic factors for ESCC. The correlation between FENDRR and miR-495-3p was evaluated by Pearson correlation analysis.

## Results

### Expression and Significance of FENDRR in Esophageal Squamous Cell Carcinoma

**Figure 1.**
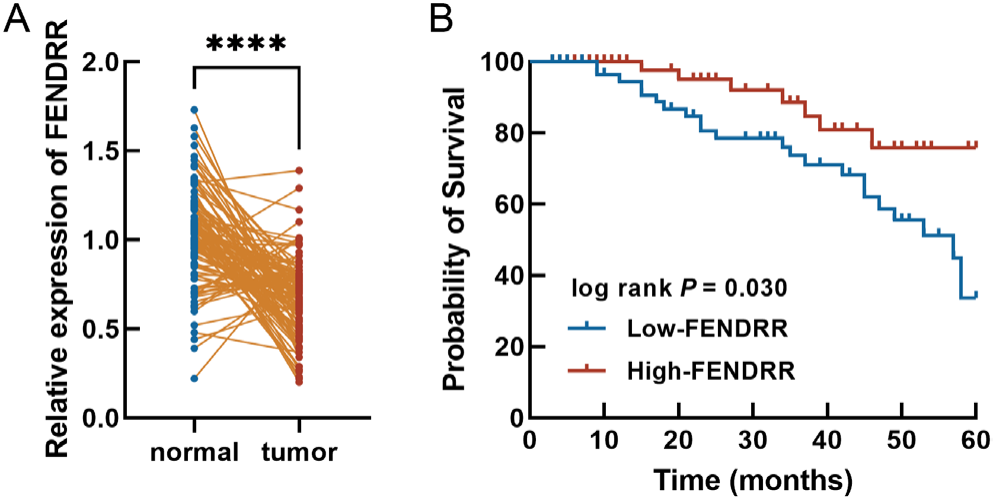
The expression of FENDRR in ESCC tissues (A) and its correlation with patients’ 5-year development-free survival (B). FENDRR was downregulated and significantly associated with the prognosis of ESCC patients. ^****^P < .0001.

In tumor tissues, FENDRR was significantly downregulated relative to the normal tissues (Figure 1A). The average FENDRR level was used as the cutoff to divide ESCC patients into the low-FENDRR with 59 patients and the high-FENDRR group with 58 patients. Esophageal squamous cell carcinoma patients with poor differentiation, advanced TNM stage, and positive lymph node metastasis were mainly included in the low-FENDRR group, and significant associations were observed in tumor size (*P* = .049), differentiation (*P* = .019), TNM stage (*P *= .007), and lymph node metastasis occurrence (*P* = .031, Table 1) with FENDRR.

Additionally, the low-FENDRR group showed a lower 5-year survival rate (Figure 1B). FENDRR (95% CI = 0.414-2.684, HR = 2.054) was identified as an independent factor for ESCC as well as TNM stage (HR = 2.684) and lymph node metastasis (HR = 2.835, Table 2).

### The Function of FENDRR in Esophageal Squamous Cell Carcinoma Cellular Processes and the Involvement of miR-495-3p

**Figure 2.**
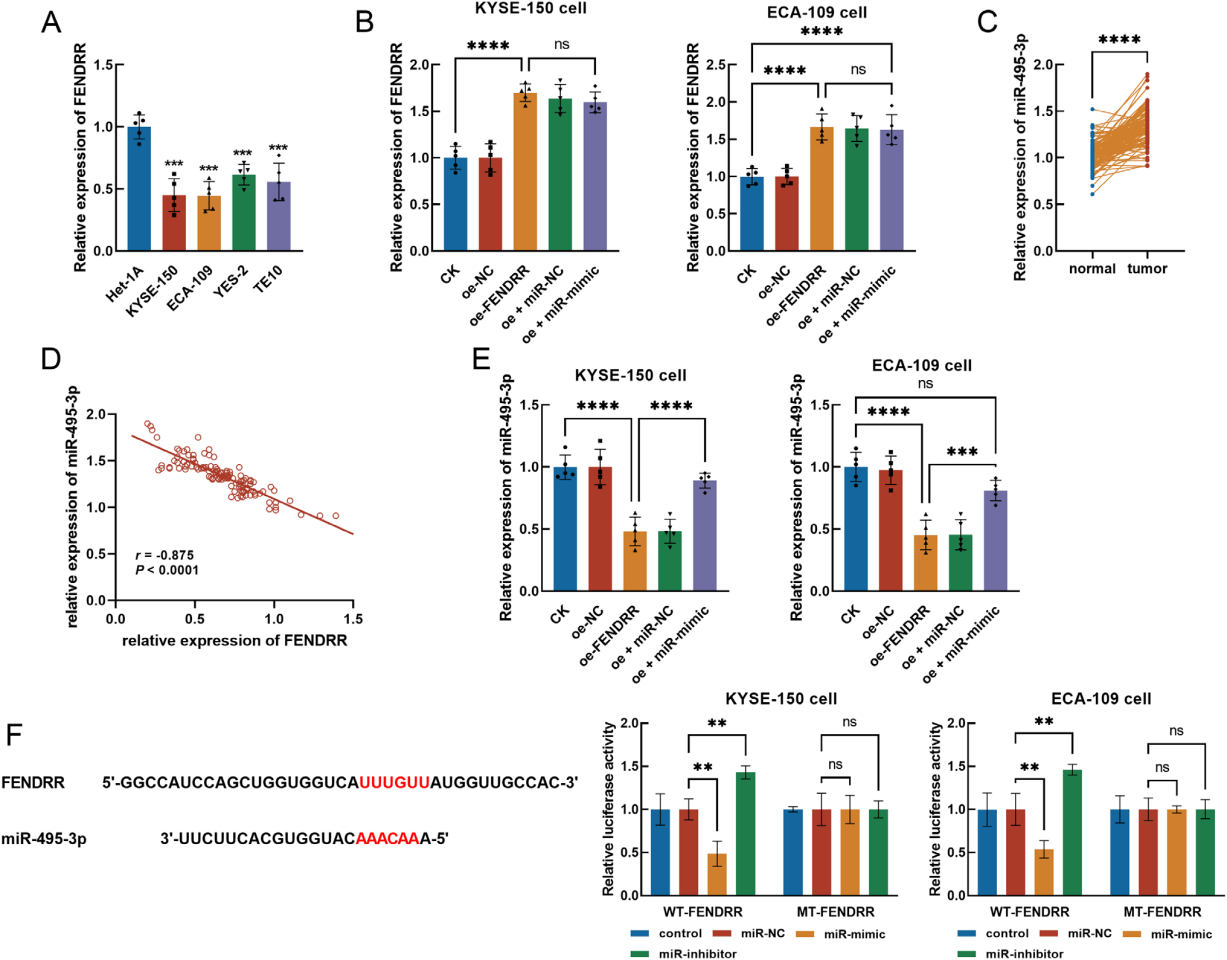
The expression of FENDRR in ESCC cells (A) and its regulation by cell transfection (B). The expression of miR-495-3p in tumor tissues (C) and its correlation with FENDRR (D). The regulatory effect of FENDRR on the expression of miR-495-3p (E). The binding sites between FENDRR and miR-495-3p and validation of their targeting relationship by the luciferase reporter assay (F). FENDRR was downregulated in ESCC cells. Overexpressing FENDRR could significantly suppress miR-495-3p. ^ns^*P* > .05, ^**^P < .01, ^***^P < .001, ^****^P < .0001.

Reduced FENDRR levels were also observed in ESCC cells compared with normal cells (Figure 2A). KYSE-150 and ECA-109 were more sensitive to the dysregulation of FENDRR and were selected for the assessment of cellular processes.

FENDRR was overexpressed in KYSE-150 and ECA-109 cells by cell transfection (Figure 2B). The miR-495-3p was predicted as a ceRNA of FENDRR from the lncRNASNP 3.0 database. Significant upregulation of miR-495-3p was observed in tumor tissues (Figure 2C), which showed a significantly negative correlation with the expression of FENDRR (*r* = −0.875, Figure 2D). Overexpressing FENDRR dramatically reduced the expression of miR-495-3p in KYSE-150 and ECA-109 cells (Figure 2E). Their binding sites were also obtained, and the targeting relationship was confirmed by the negatively regulated luciferase activity of FENDRR by miR-495-3p in KYSE-150 and ECA-109 cells (Figure 2F). The miR-495-3p mimic has not affected FENDRR expression (Figure 2B) but dramatically reversed the inhibited miR-495-3p by FENDRR (Figure 2E).

**Figure 3.**
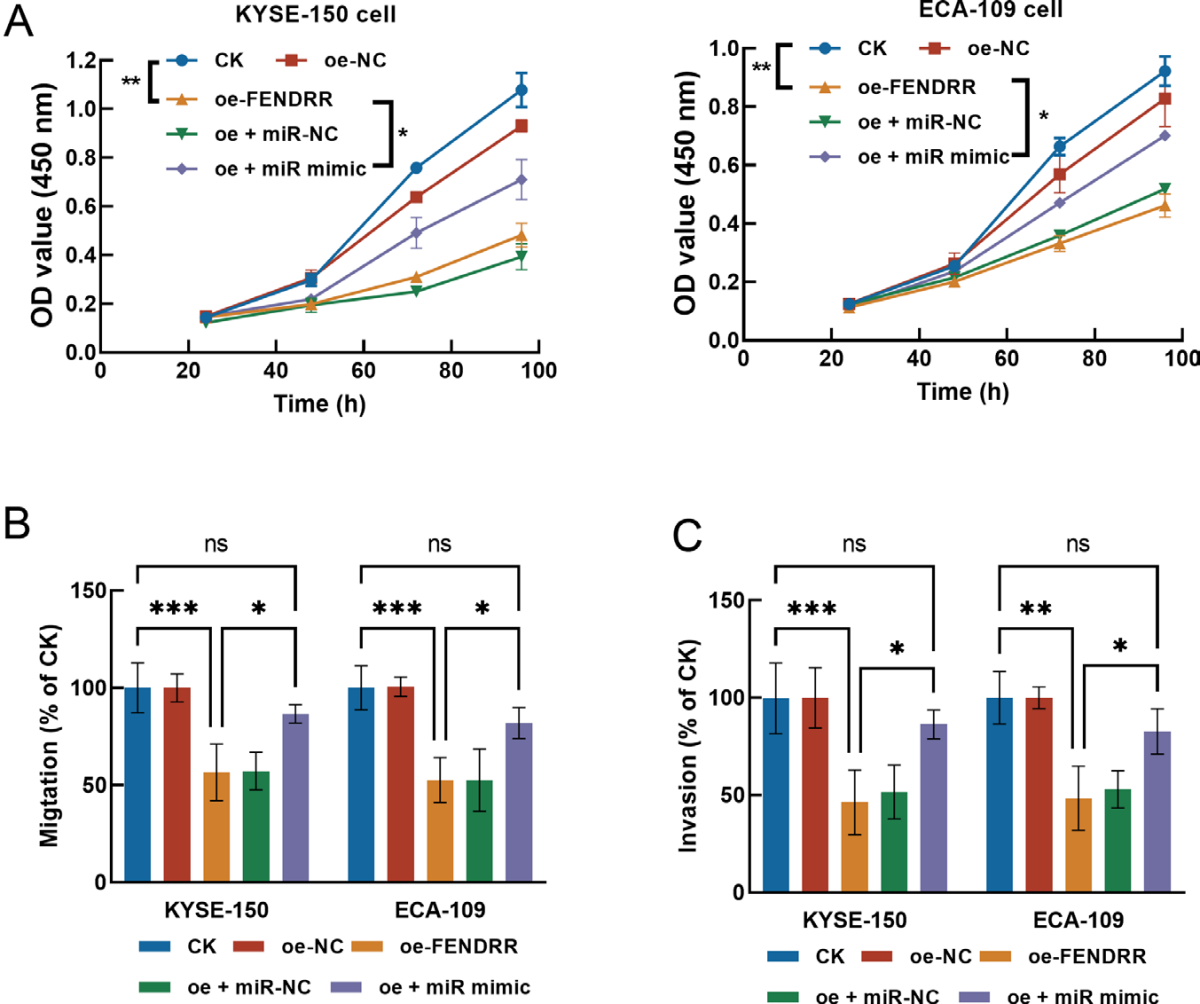
The effect of FENDRR on the proliferation (A), migration (B), and invasion (C) of KYSE-150 and ECA-109 cells, and the involvement of miR-495-3p. Overexpressing FENDRR significantly inhibited cell growth and motility, which was reversed by the overexpression of miR-495-3p. ^ns^*P* > .05, ^*^P < .05, ^**^P < .01, ^***^P < .001.

In KYSE-150 and ECA-109 cells, elevated FENDRR decreased proliferation (Figure 3A), migration (Figure 3B), and invasion (Figure 3C) significantly. While miR-495-3p could alleviate the inhibitory effects of FENDRR, indicating its involvement in the function of FENDRR (Figure 3A-C).

## Discussion

The occurrence of esophageal carcinoma shows a significant regional difference, with ESCC is the highly prevalent subtype in Asia, especially in China.^21^ Although diagnostic and therapeutic technologies have improved a lot, the occurrence and mortality of ESCC have still increased rapidly. Previous studies revealed the significance of lncRNAs in cancer development and regulation, implying that lncRNAs are expected to serve as molecular indicators of ESCC and potential targets for tumor treatments.

In the digestive system, FENDRR was evidenced to play roles in gastric cancer, colon cancer, and hepatocellular carcinoma progression.^17^^,^^22^^,^^23^ The regulatory effect and significance of FENDRR dysregulation in its involved cancer have also been disclosed. For example, the reduced level of FENDRR in colon cancer was found to predict advanced clinical stages and adverse outcomes.^12^ The prognostic significance of FENDRR was also demonstrated in breast cancer, where FENDRR was downregulated and correlated with shorter overall and progression-free survival of patients, and associated with increasing PR, HER-2, and lymphatic metastasis.^24^ Herein, FENDRR downregulation was also observed in ESCC patients’ tumor tissues. The decreasing FENDRR showed a close association with the severity of ESCC patients, behaving as the larger tumor size, poorer differentiation, advanced TNM stage, and positive lymph node metastasis. Moreover, FENDRR also served as a prognostic indicator that showed significant association with the 5-year development-free survival of ESCC patients.

Recently, lncRNAs have been considered essential regulators in malignant tumors that regulate various cellular functions, including cell motility, stemness, and differentiation.^25^ The proliferation of tumor cells is associated with tumor growth and therefore indirectly affects the development of cancers.^26^ Cell motility is an important factor related to cancer metastasis.^27^ FENDRR was previously reported to regulate the migratory, invasive, and growth capacities of cholangiocarcinoma, osteosarcoma, prostate cancer, and renal carcinoma cells, evidencing its tumor regulator roles.^28^^-^^31^ Herein, ESCC cells also showed significant downregulation of FENDRR. Overexpressing FENDRR was found to inhibit the growth and metastasis ability of ESCC cells, suggesting its potential tumor suppressor role in ESCC progression.

Long non-coding RNAs could modulate the expression and effect of functional miRNAs and therefore further regulate tumor progression.^32^ The miR-495-3p was negatively regulated by FENDRR. The cellular processes of various cancers were previously shown to be regulated by miR-495-3p. For instance, miR-495-3p was observed to inhibit colorectal cancer cells, which depressed cell growth and migration and therefore was identified as a tumor suppressor.^33^ A previous miRNA profile revealed upregulated miR-495-3p in post-ablation neosquamous mucosa.^34^ In esophageal cancer, miR-495-3p was demonstrated to mediate the effect of FAM83A-AS1.^20^ Here, overexpressing miR-495-3p attenuated the inhibitory effect of FENDRR on ESCC cell growth and motility, indicating its involvement in the regulation of ESCC cellular processes by FENDRR.

However, the subjects of the present study were enrolled from a single center, and the sample size is not large enough, which might limit the clinical results. Therefore, future studies should include multiple centers and expanded sample size. On the other hand, the underlying molecular mechanisms also need further investigation. Targeting the 3’ UTR of mRNAs has been widely accepted as the major regulatory mechanism of miRNAs. Previous studies have reported miR-495-3p could target CDK1, cadherin 2, and HMGB1 to display its functional role in various cancers, and BUB1 was demonstrated as the direct target of miR-495-3p in esophageal carcinoma.^33^^,^^35^^-^^37^ Further mechanism studies are needed to complete the regulatory axis of FENDRR and to provide more potential targets for the clinical therapy of ESCC.

Taken together, the present study confirmed the downregulation of FENDRR in ESCC, which predicted the severity and unfavorable outcomes of patients. Overexpressing FENDRR dramatically prevented ESCC cell growth and motility via miR-495-3p.

## Figures and Tables

**Table 1.** Association of FENDRR Expression with Patients’ Clinicopathological Features

	Cases (n = 117)	Low-FENDRR (n = 59)	High-FENDRR (n = 58)	*P*
Age				.636
<60	53	28	25	
≥60	64	31	33	
Gender				.277
Male	75	35	40	
Female	42	24	18	
Tumor location				.108
Upper and middle	68	30	38	
Lower	49	29	20	
Tumor size				.049
<4	66	28	38	
≥4	51	31	20	
Differentiation				.019
Well-moderate	81	35	46	
Poor	36	24	12	
TNM stage				.007
I-II	79	33	46	
III	38	26	12	
Lymph node metastasis				.031
Absent	82	36	46	
Present	35	23	12	

**Table 2.** Multivariate Cox Regression Analysis Evaluating the Prognostic Value of Patients’ Clinicopathological Features

	95%CI	HR	*P*
FENDRR	0.414-2.684	2.054	.012
Age	0.522-3.306	1.313	.563
Gender	0.450-3.154	1.192	.724
Tumor location	0.693-4.416	1.750	.236
Tumor size	0.597-3.847	1.516	.381
Differentiation	0.752-4.799	1.900	.175
TNM stage	1.066-6.756	2.684	.036
Lymph node metastasis	1.104-7.283	2.835	.030

## Data Availability

The data that support the findings of this study are available on request from the corresponding author.

## References

[1] Huang FL Yu SJ. Esophageal cancer: risk factors, genetic association, and treatment. Asian J Surg. 2018;41(3):210 215. (doi: 10.1016/j.asjsur.2016.10.005) 27986415

[2] Lai RP. Esophageal squamous cell carcinoma: time to predict the risk? Am J Gastroenterol. 2021;116(8):1757 1758. (doi: 10.14309/ajg.0000000000001293) 33941748

[3] Lam AK. Introduction: esophageal squamous cell carcinoma-current status and future advances. Methods Mol Biol. 2020;2129:1 6. (doi: 10.1007/978-1-0716-0377-2_1) 32056165

[4] Niu C Liu Y Wang J Risk factors for esophageal squamous cell carcinoma and its histological precursor lesions in China: a multicenter cross-sectional study. BMC Cancer. 2021;21(1):1034. (doi: 10.1186/s12885-021-08764-x) PMC8444572 34530751

[5] Uhlenhopp DJ Then EO Sunkara T Gaduputi V. Epidemiology of esophageal cancer: update in global trends, etiology and risk factors. Clin J Gastroenterol. 2020;13(6):1010 1021. (doi: 10.1007/s12328-020-01237-x) 32965635

[6] Zhou N Rajaram R Hofstetter WL. Management of locally advanced esophageal cancer. Surg Oncol Clin N Am. 2020;29(4):631 646. (doi: 10.1016/j.soc.2020.06.003) 32883463

[7] Waters JK Reznik SI. Update on management of squamous cell esophageal cancer. Curr Oncol Rep. 2022;24(3):375 385. (doi: 10.1007/s11912-021-01153-4) 35142974

[8] Li Y Yang B Ma Y Phosphoproteomics reveals therapeutic targets of esophageal squamous cell carcinoma. Signal Transduct Target Ther. 2021;6(1):381. (doi: 10.1038/s41392-021-00682-5) PMC8585941 34764241

[9] Yan H Bu P. Non-coding RNA in cancer. Essays Biochem. 2021;65(4):625 639. (doi: 10.1042/EBC20200032) 33860799 PMC8564738

[10] Li W Liu J Zhao H. Identification of a nomogram based on long non-coding RNA to improve prognosis prediction of esophageal squamous cell carcinoma. Aging (Albany NY). 2020;12(2):1512 1526. (doi: 10.18632/aging.102697) 31978896 PMC7053640

[11] Shi X Liu X Pan S A novel autophagy-related long non-coding RNA signature to predict prognosis and therapeutic response in esophageal squamous cell carcinoma. Int J Gen Med. 2021;14:8325 8339. (doi: 10.2147/IJGM.S333697) 34815705 PMC8605829

[12] Liu J Du W. LncRNA FENDRR attenuates colon cancer progression by repression of SOX4 protein. Onco Targets Ther. 2019;12:4287 4295. (doi: 10.2147/OTT.S195853) 31213846 PMC6549791

[13] Ma J Zhao G Du J Li J Lin G Zhang J. LncRNA FENDRR inhibits gastric cancer cell proliferation and invasion via the miR-421/SIRT3/Notch-1 axis. Cancer Manag Res. 2021;13:9175 9187. (doi: 10.2147/CMAR.S329419) 34938121 PMC8685553

[14] Pan H Yu T Sun L Chai W Liu X Yan M. LncRNA FENDRR-mediated tumor suppression and tumor-immune microenvironment changes in non-small cell lung cancer. Transl Cancer Res. 2020;9(6):3946 3959. (doi: 10.21037/tcr-20-2147) 35117761 PMC8797579

[15] Cheng C Li H Zheng J Xu J Gao P Wang J. FENDRR sponges miR-424-5p to inhibit cell proliferation, migration and invasion in colorectal cancer. Technol Cancer Res Treat. 2020;19:1533033820980102. (doi: 10.1177/1533033820980102) 33356998 PMC7768317

[16] Gong L Zhu L Yang T. Fendrr involves in the pathogenesis of cardiac fibrosis via regulating miR-106b/SMAD3 axis. Biochem Biophys Res Commun. 2020;524(1):169 177. (doi: 10.1016/j.bbrc.2020.01.062) 31982134

[17] Qian G Jin X Zhang L. LncRNA FENDRR upregulation promotes hepatic carcinoma cells apoptosis by targeting miR-362-5p via NPR3 and p38-MAPK pathway. Cancer Biother Radiopharm. 2020;35(9):629 639. (doi: 10.1089/cbr.2019.3468) 32251605

[18] Zhang G Wang Q Zhang X Ding Z Liu R. LncRNA FENDRR suppresses the progression of NSCLC via regulating miR-761/TIMP2 axis. Biomed Pharmacother. 2019;118:109309. (doi: 10.1016/j.biopha.2019.109309) 31545237

[19] Zhu Y Zhang X Wang L FENDRR suppresses cervical cancer proliferation and invasion by targeting miR-15a/b-5p and regulating TUBA1A expression. Cancer Cell Int. 2020;20:152. (doi: 10.1186/s12935-020-01223-w) PMC7204253 32398968

[20] Huang GM Zang HL Geng YX Li YH. LncRNA FAM83A-AS1 aggravates the malignant development of esophageal cancer by binding to miR-495-3p. Eur Rev Med Pharmacol Sci. 2020;24(18):9408 9415. (doi: 10.26355/eurrev_202009_23024) 33015782

[21] Mehryar MM Li SY Liu HW Prevalence of human papillomavirus in esophageal carcinoma in Tangshan, China. World J Gastroenterol. 2015;21(10):2905 2911. (doi: 10.3748/wjg.v21.i10.2905) 25780287 PMC4356909

[22] Luo T Zhao J Lu Z Characterization of long non-coding RNAs and MEF2C-AS1 identified as a novel biomarker in diffuse gastric cancer. Transl Oncol. 2018;11(5):1080 1089. (doi: 10.1016/j.tranon.2018.06.007) 30005210 PMC6067087

[23] Yin SL Xiao F Liu YF Chen H Guo GC. Long non-coding RNA FENDRR restrains the aggressiveness of CRC via regulating miR-18a-5p/ING4 axis. J Cell Biochem. 2020;121(8-9):3973 3985. (doi: 10.1002/jcb.29555) 31724220

[24] Li Y Zhang W Liu P Long non-coding RNA FENDRR inhibits cell proliferation and is associated with good prognosis in breast cancer. Onco Targets Ther. 2018;11:1403 1412. (doi: 10.2147/OTT.S149511) 29559798 PMC5857152

[25] Agostini M Mancini M Candi E. Long non-coding RNAs affecting cell metabolism in cancer. Biol Direct. 2022;17(1):26. (doi: 10.1186/s13062-022-00341-x) PMC9526990 36182907

[26] Martínez-Reyes I Chandel NS. Cancer metabolism: looking forward. Nat Rev Cancer. 2021;21(10):669 680. (doi: 10.1038/s41568-021-00378-6) 34272515

[27] Stuelten CH Parent CA Montell DJ. Cell motility in cancer invasion and metastasis: insights from simple model organisms. Nat Rev Cancer. 2018;18(5):296 312. (doi: 10.1038/nrc.2018.15) 29546880 PMC6790333

[28] Qin X Lu M Zhou Y Li G Liu Z. LncRNA FENDRR represses proliferation, migration and invasion through suppression of survivin in cholangiocarcinoma cells. Cell Cycle. 2019;18(8):889 897. (doi: 10.1080/15384101.2019.1598726) 30983519 PMC6527288

[29] Kun-Peng Z Xiao-Long M Chun-Lin Z. LncRNA FENDRR sensitizes doxorubicin-resistance of osteosarcoma cells through down-regulating ABCB1 and ABCC1. Oncotarget. 2017;8(42):71881 71893. (doi: 10.18632/oncotarget.17985) 29069754 PMC5641097

[30] Zhang YQ Chen X Fu CL FENDRR reduces tumor invasiveness in prostate cancer PC-3 cells by targeting CSNK1E. Eur Rev Med Pharmacol Sci. 2019;23(17):7327 7337. (doi: 10.26355/eurrev_201909_18838) 31539119

[31] He W Zhong G Wang P Jiang C Jiang N Huang J. Downregulation of long noncoding RNA FENDRR predicts poor prognosis in renal cell carcinoma. Oncol Lett. 2019;17(1):103 112. (doi: 10.3892/ol.2018.9624) 30655744 PMC6313193

[32] Panni S Lovering RC Porras P Orchard S. Non-coding RNA regulatory networks. Biochim Biophys Acta Gene Regul Mech. 2020;1863(6):194417. (doi: 10.1016/j.bbagrm.2019.194417) 31493559

[33] Zhang JL Zheng HF Li K Zhu YP. miR-495-3p depresses cell proliferation and migration by downregulating HMGB1 in colorectal cancer. World J Surg Oncol. 2022;20(1):101. (doi: 10.1186/s12957-022-02500-w) PMC8966301 35354479

[34] Sreedharan L Mayne GC Watson DI MicroRNA profile in neosquamous esophageal mucosa following ablation of Barrett’s esophagus. World J Gastroenterol. 2017;23(30):5508 5518. (doi: 10.3748/wjg.v23.i30.5508) 28852310 PMC5558114

[35] Yang H Chen XW Song XJ Du HY Si FC. Baitouweng decoction suppresses growth of esophageal carcinoma cells through miR-495-3p/BUB1/STAT3 axis. World J Gastrointest Oncol. 2024;16(7):3193 3210. (doi: 10.4251/wjgo.v16.i7.3193) 39072160 PMC11271792

[36] Song C Wang Q Qi Q MiR-495-3p regulates myoblasts proliferation and differentiation through targeting cadherin 2. Anim Biotechnol. 2023;34(7):2617 2625. (doi: 10.1080/10495398.2022.2109042) 35951546

[37] Tang J Pan H Wang W MiR-495-3p and miR-143-3p co-target CDK1 to inhibit the development of cervical cancer. Clin Transl Oncol. 2021;23(11):2323 2334. (doi: 10.1007/s12094-021-02687-6) 34387848

